# The Influence of Self-Heating Iron on the Thermal, Mechanical, and Swelling Properties of PDMS Composites for Organic Solvents Removal

**DOI:** 10.3390/polym13234231

**Published:** 2021-12-02

**Authors:** Mohamed S. A. Darwish, Laila M. Al-Harbi

**Affiliations:** 1 Egyptian Petroleum Research Institute, 1 Ahmed El-Zomor Street, El Zohour Region, Nasr City, Cairo 11727, Egypt; 2 Chemistry Department, Faculty of Science, King Abdul-Aziz University, P.O. Box 80203, Jeddah 21589, Saudi Arabia; lalhrbi@kau.edu.sa

**Keywords:** self-heating iron, PDMS, VOC, thermal stability, swelling, mechanical properties

## Abstract

Volatile organic compounds pollute the environment and pose a serious threat to human health due to their toxicity, mutagenicity, and carcinogenicity. In this context, it is highly desirable to fabricate high-performance poly (dimethylsiloxane) (PDMS) composites to remove organic solvents from the environment using a simple technique. Therefore, in the present study, Fe-PDMS composites were fabricated using a technique based on magnetic induction heating with iron particles serving as a self-heating agent. Under an alternating magnetic field, the iron particles served as a thermal source that assisted in the progression of PDMS crosslinking. The influence of self-heating iron on the properties of the fabricated Fe-PDMS composites was also investigated. The hydrosilation reaction occurring during the crosslinking process was controlled using FT-IR. The heating efficiency of PDMS 1, PDMS 2, and PDMS 3 was studied as the function of induction time (0–5 min) and the function of iron content (0%, 1%, and 30% wt.%). The results revealed that the mechanical properties of the PDMS 2 composite were enhanced compared to those of the PDMS 1 and PDMS 3 composites. The mechanical properties of PDMS 3 were the least efficient due to cluster formation. PDMS 3 exhibited the highest thermal stability among all composites. Furthermore, the swelling behavior of different materials in various organic solvents was studied. PDMS was observed to swell to the greatest extent in chloroform, while swelling to a large extent was observed in toluene, pentane, and petroleum ether. PDMS swelling was the least in n-butanol. The elastomeric behavior of crosslinked PDMS, together with its magnetic character, produces stimuli-responsive magneto-rheological composites, which are quite efficient and suitable for applications involving the removal of organic solvents.

## 1. Introduction

Silicones exhibit a combination of the properties of two types of materials: organic and inorganic. Therefore, silicones are considered suitable polymeric matrices for various inorganic powders [1,2,3]. Polydimethylsiloxane (PDMS) is a biocompatible, inexpensive, and convenient-to-use elastomer comprising an inorganic siloxane (Si-O) backbone and organic methyl groups attached to the constituent silicon atoms [4,5,6]. The perfect PDMS system includes silanol-terminated PDMS, a crosslinker, and a catalyst as the major components, while reinforcing fillers and other additives may be added according to the application. Such systems combine the properties induced by the presence of metal with those specific to polymers, including the property of easy processability, which allows for the possibility of developing a wide range of composite materials with desired properties [7,8,9]. PDMS exhibits matchless behaviors in terms of elasticity, thermal stability, and mechanical, chemical, and optical properties [10,11,12]. It has attracted particular interest as it has the lowest surface energy reported so far, along with a hydrophobic character. An appropriate novel composite selection and layout would allow altering the properties of products according to the demands and future applications such as magneto-rheological fluids and electromagnetic wave shields [13,14,15]. PDMS has several applications, such as theranostics and drug delivery in biomedical applications [16]. The Ag-PDMS composites have been used in electrical and optical devices [17]. Soft materials containing a polymeric matrix with iron particles incorporated in it are of high significance for use in stimuli-responsive magneto-rheological composites [18,19]. The impact of iron as self-heating particles (5, 10, 15% volume fraction) in the curing control of PDMS composites was investigated. The fabrication of thermally stable Fe-PDMS composite revealed promising results for the use of this composite as a thermal composite in applications where higher heat resistance is required [12]. The isotropic distribution of iron filler particles (30% volume fraction) was observed without any influence from the magnetic field during magneto-rheological elastomer (MRE) composites’ preparation. The anisotropic arrangement of iron fillers within the MRE composites was observed in the presence of a magnetic field during fabrication. It was shown that the linear arrangement of the iron particle chain induced magnetization within the composite [18]. For magnetic elastomers, pre-aligning the magnetic particles within the polymer matrix proved to be a successful approach to improving their responsiveness to external magnetic fields, showing a higher degree of deformability and even anisotropic mechanical response [9]. Among the scope of efficient magnetic particles, the most distinguished MR material is the carbonyl iron particle (CI). However, serious sedimentation and agglomeration problems due to the large density mismatch between the CI particle and medium oil have limited its engineering applications [10]. Drainage of organic compounds to surface waters threatens the aquatic environment with effects such as acute and chronic toxicity in the aquatic organisms, accumulation of pollutants in the marine ecosystem, and loss of habitats and biodiversity of marine life [3,4,5]. Petroleum hydrocarbon compounds, including toluene, chloroform, pentane, petroleum ether, and n-butanol, are often drained into the surface and underground waters due to industrial activities, particularly the mishandling of petrochemicals, reservoir leakages, and inappropriate waste disposal practices [6,7,8]. Chemical contamination of water has caused water pollution to remain a serious environmental and public health problem. PDMS is notably hydrophobic, and water is unable to readily permeate its surface. On the contrary, most organic solvents can permeate the PDMS surface, which is the basis of their application in solvent removal. PDMS is highly soluble in non-polar solvents and, therefore, to render it chemically stable, the crosslinking of PDMS is necessary. PDMS sponge/carbon nanotubes are more comprehensive than the pure PDMS for efficient removal of oil from immiscible oil–water mixtures [4]. PDMS/graphene aerogels displayed a high adsorption capacity toward various organic solvents in the range of 48–96 g/g [5]. PDMS-based hydrophobic sorbents were fabricated via bulk polymerization of tetraethyl orthosilicate and different lengths of PDMS, without using any activator, initiator, catalyst, or solvent. The prepared sorbents have a high swelling ability in organic liquids/oils in the range of 9–21 g/g [7]. Mazzotta et al. proposed the production of reusable magnetic nanocomposites using PDMS/multiwalled carbon nanotubes and utilized them as absorbents to selectively collect oil from oily wastewater [11]. The hydrosilylation reaction may be accelerated by applying heat, ultraviolet (UV) irradiation, or gamma-ray irradiation. Each fabricating method has its characteristic advantages and disadvantages. The curing condition for pristine PDMS is approximately 48 h at room temperature or 10 min at a high temperature (150 °C). UV-based curing may be applied to avoid exposure to high curing temperatures, although the overall penetration of the UV-cured system is low compared to heat-cured systems. The magnetic induction heating technique appears promising as a facile technique to overcome the following shortcomings of the existing methods of fabrication: (1) the self-heating iron particles serve as an internal thermal source dispersed homogenously in the PDMS matrix, which allows effective curing due to the enclosure of a steady heat allocation throughout the crosslinking process, which is not the case with the UV-cured system. (2) The presence of iron particles enhances the reactivity of the hydrosilation process, thereby allowing the completion of the curing process in a shorter duration compared to the heat-cured system. (3) The magnetic induction heating technique allows controlling the curing process as it is possible to tune the curing rate by altering the iron content and induction time. (4) PDMS is degraded at a low temperature and, therefore, fails to meet the service requirement for heat-resistant elastomers. The presence of iron in the PDMS matrix, however, enhances the thermal stability of the composite. (5) The magnetic character is advantageous for reuse as the composites could be separated from the medium simply by using a magnetic source. Therefore, the present study aimed to investigate the performance of the PDMS composite in the removal of organic solvents. The study involved continuing the deep investigation of the effect of utilizing iron particles as a self-heating agent using the magnetic induction heating technique on the properties of PDMS composites. The curing process was followed using Fourier transform infrared (FTIR), and the composites were analyzed for the distribution of the filler within the material using scanning electron microscopy (SEM). The mechanical properties and the thermal stability of the materials were investigated using dynamic mechanical analysis (DMA) and thermogravimetric analysis (TGA), respectively. Finally, the compatibility and the swelling behavior in the presence of different organic solvents were evaluated.

## 2. Materials and Methods

### 2.1. Materials

PDMS Sylgard 184 was supplied in a two-component kit containing a pre-polymer (silicone elastomer base) and a crosslinker (184 silicone elastomer curing agent) in a ratio of 10:1 by weight (Dow Corning Corporation, Midland, MI, USA). Iron powder (size 5–9 µm; CAS no. 7439-89-6) was purchased from Sigma-Aldrich (St. Louis, MO, USA).

### 2.2. Fabrication of PDMS Composites Using the Magnetic Induction Heating System

**Figure 1:**
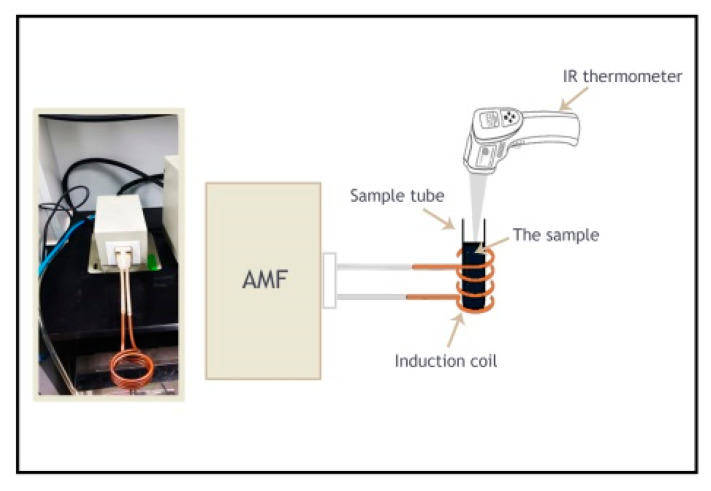
The magnetic induction heating system.

Fe-PDMS composites PDMS 1, PDMS 2, and PDMS 3 were fabricated by adding PDMS to iron powder in a proportion of 0%, 1%, and 30% (wt.%), respectively. The mixture was stirred uniformly while applying a vacuum to remove bubbles. Since magnetic stirring is not recommended for the dispersion of magnetic particles, mechanical stirring at 1000 rpm was applied for two min. The hydrosilation process was allowed to occur for 5 min using magnetic induction heating with a frequency of 142 kHz and 1 kW of power. The temperature increases were recorded using an infrared thermometer (Figure 1).

### 2.3. Characterizations

The thermogravimetric analysis (TGA) was conducted using Q500 (TA instruments, Elstree, UK). The weight loss was recorded at different temperatures (25–900 °C; rate = 10 °C/min under nitrogen). The mechanical properties were measured by performing the dynamic mechanical analysis (DMA) using Q800. The DMA experiments were conducted in the temperature range of 30–200 °C and a heating rate of 5 °C/min (TA Instruments, Elstree, UK). A Bruker Tensor 27 Infrared Spectrometer (Bruker, USA) was employed for Fourier Transform Infrared (FTIR). The electron microscopy observation was performed under FE-SEM (Zeiss ULTRA Plus equipped with a Schottky cathode), and the images were obtained using Smart SEM v5.05 software operated at 1.5 kV (Zeiss, Oberkochen, Germany). The dimension of the samples used in the analysis measurements was 2 ± 0.1 mm in width and 0.2 ± 0.08 mm in thickness. The swelling behavior was investigated in different organic solvents: toluene, chloroform, pentane, petroleum ether, and n-butanol. The weight of the fabricated PDMS was recorded prior to immersion into the solvent, as depicted in Equation (1). Subsequently, after the removal of samples from the solvent (after 30 min) followed by rapid drying, the weight gain in the PDMS samples was recorded. The weights were measured on a mass balance.

(1) $$\mathrm{Degree}\;\mathrm{of}\;\mathrm{swelling}\;(S)=\frac{wt1-wt2}{wt1\;}$$

Here, *wt*1 and *wt*2 denote the weight of the material prior to and after immersion into the solvent, respectively.

## 3. Results and Discussion

### 3.1. Fabrication of the PDMS Composites

**Figure 2:**
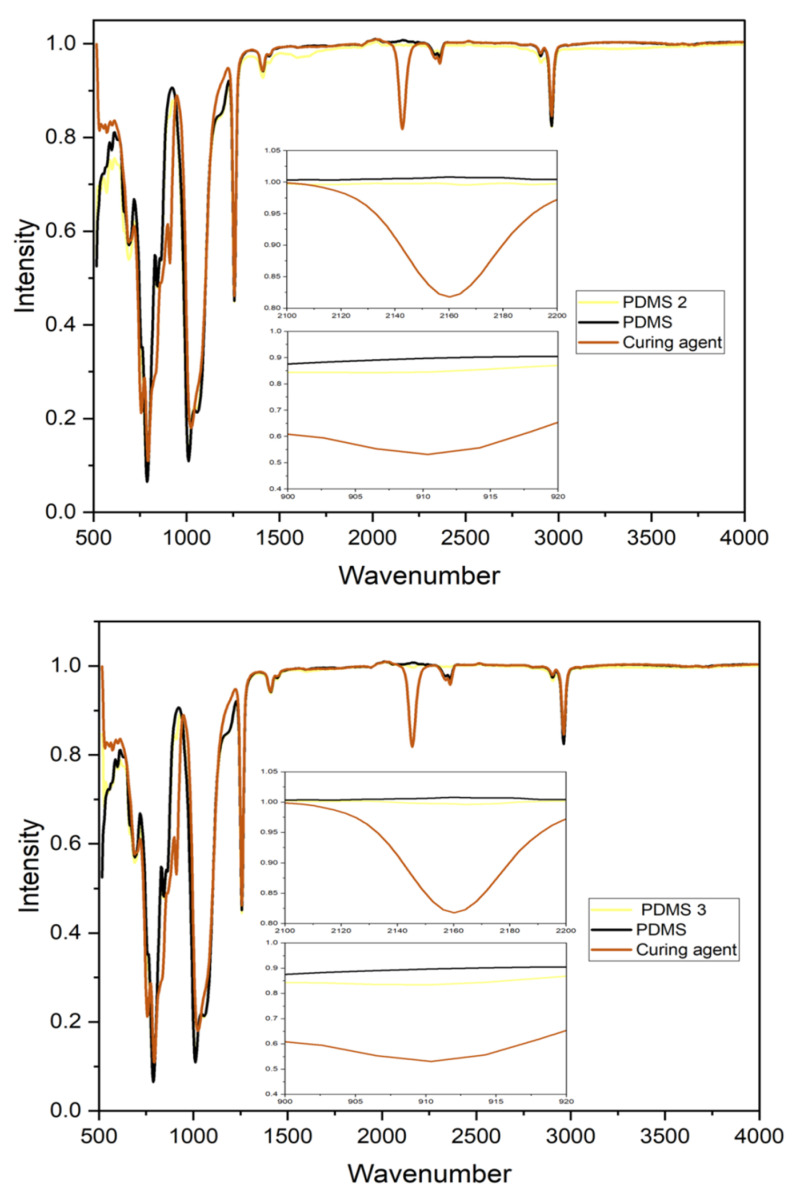
FT-IR of PDMS 2 and PDMS 3.

In general, to confer chemical stability to the PDMS composites, a three-dimensional crosslinked net has to be included in their structures. This is achieved through a reaction between the vinyl-end functional groups on the linear PDMS chains and a multifunctional crosslinker in the presence of a catalyst. Since the structure of crosslinked PDMS materials is usually fabricated using the hydrosilation reaction, factors such as the hydrosilation rate, the vinyl groups of the PDMS structure, the number of Si-H groups in the structure of the crosslinker, and the type and molecular structure of the catalytic complex become important. In the present study, Fe-PDMS composites were fabricated using the magnetic induction heating technique. Under an alternating current (AC) magnetic field, iron particles in the composite structure served as micro-heating agents due to the relaxation phenomena occurring as a result of the magnetization-based internal rotation of the iron particles in a viscous PDMS medium. The crosslinking process alters the characteristic of the pre-polymer from viscous to elastic. The curing process may be accelerated by increasing the temperature [20]. After the completion of the crosslinking process, the PDMS composite exhibits a rubbery appearance. Material performance is determined to a great extent by the framework of the crosslinked net and the residue groups in the bulk and interface of the material. Therefore, it is of great importance to monitor the reaction throughout the crosslinking process and to distinguish the crosslinked networks being fabricated. The process to obtain a cured polymer is based on the reaction between the Si–H crosslinker group and the (Si–CH=CH_2_ pristine polymer group [21]). According to the FT-IR results (Figure 2), the characteristic bands of PDMS did not shift upon increasing Fe particles and were, therefore, concentration-independent. This result suggested that no chemical reaction occurred between the Fe particles and PDMS during the fabrication process. The influence of iron particles serving as micro-heating agents on the crosslinking matrix was also studied using the FT-IR analysis. It was observed that after the crosslinking, the (–Si–H) peaks had disappeared. The characteristic bands corresponding to the bending and stretching of (–Si–H) appear at 910 cm^−1^ and 2160 cm^−1^, and their appearance indicates the presence of the crosslinker in the structure during the curing process (Figure 2). The crosslinker agent also exhibited reduced (–Si–H) in the integration region, indicating that (–Si–H) was being used throughout the fabrication process. Moreover, the band was almost linear as the entire crosslinker agent was consumed in the fabrication process (Figure 2) [12]. In contrast, PDMS 1 exhibited no change in the crosslinker peak due to the absence of iron particles in the system. The crosslinking process has been conducted previously at different temperatures, and the best crosslinking conditions for obtaining pristine PDMS are reported to be approximately 10 min of reaction at 150 °C, 45 min of reaction at 100 °C, or two days of reaction at room temperature [7,8]. Two mechanisms are available for stimulating the process of hydrosilation: the platinum-catalyzed mechanism and the radical chain addition mechanism [8]. It is also reported that the curing of PDMS could be conducted using a high-temperature vulcanization process, in which the polymerization is initiated by the decomposition of a free radical initiator (such as an organic peroxide) at temperatures of over 100 °C [8,11]. In the system fabricated in the present study, the presence of iron particles enhanced the reactivity of the hydrosilation process, thereby allowing the curing process to occur in a shorter duration without requiring the use of a free radical initiator or a catalyst.

### 3.2. Heating Efficiency

**Figure 3:**
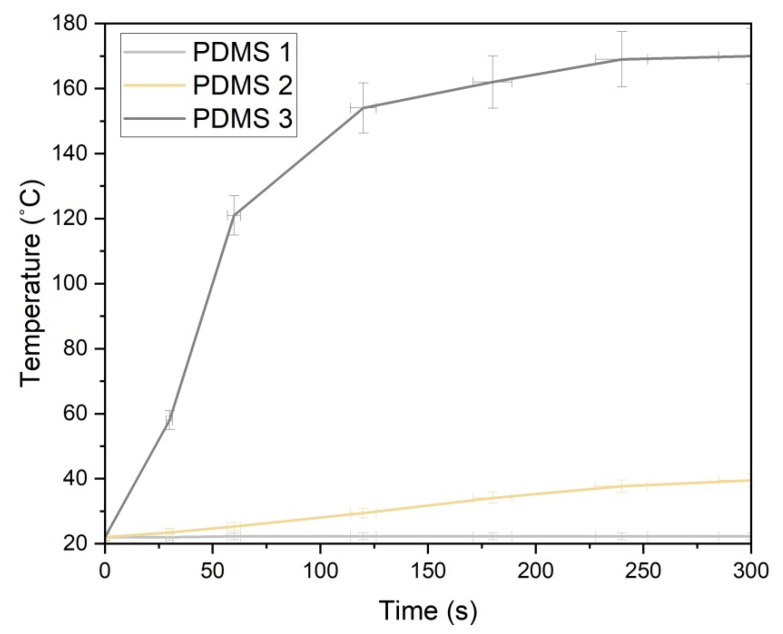
The heating efficiency of PDMS 1, PDMS 2, and PDMS 3.

The hydrosilation reaction involves the reaction of the crosslinker with the pre-polymer to produce a cured polymer. The heating efficiency of the crosslinker and the pre-polymer mixture was investigated based on the presence of iron particles in the PDMS matrix using a magnetic induction heating system. Uniform distribution of iron particles in PDMS is desired to ensure a steady heat allocation throughout the crosslinking process. Iron was incorporated in the PDMS matrix in the concentrations of 0%, 1%, and 30% (wt.%) for different exposure durations (0–5 min) at a frequency of 142 kHz and 1 kW of power. A quasi-adiabatic reign, in which no heat exchange occurs between the sample and the surroundings, was assumed. As illustrated in Figure 3, for PDMS 3, the temperature elevated linearly with time during the initial stage (up to 60 s), and then the increase in the temperature decelerated gradually until the saturation stage was reached. Afterward, the temperature did not increase any further, as the thermal equilibrium had been attained, a stage at which the heating rate equals the cooling rate.

As visible in Figure 3, with the increase in the iron content, the heating rate also increased, and the samples were heated faster by the magnetic field. PDMS 3 exhibited the highest heating efficiency under the same magnetic field. The maximum temperature reached after 5 min was 22.3 °C, 39.5 °C, and 170 °C for PDMS 1, PDMS 2, and PDMS 3, respectively. With increasing iron content, the heating rate also increased, causing the material to be heated promptly with the applied magnetic field. The heating rates achieved were 0.03 °C/s and 1.2 °C/s for PDMS 2 and PDMS 3, respectively. No increase in temperature was observed in PDMS 1 due to the absence of iron particles. The heating efficiency of iron particles under an applied magnetic field is a consequence of two loss mechanisms: Néel relaxation and Brownian relaxation [22,23]. While Néel relaxation is affected by interparticle dipole/dipole interactions, Brownian relaxation is associated with the rotation of the iron particles in a viscous PDMS medium.

### 3.3. Thermal Stability

**Figure 4:**
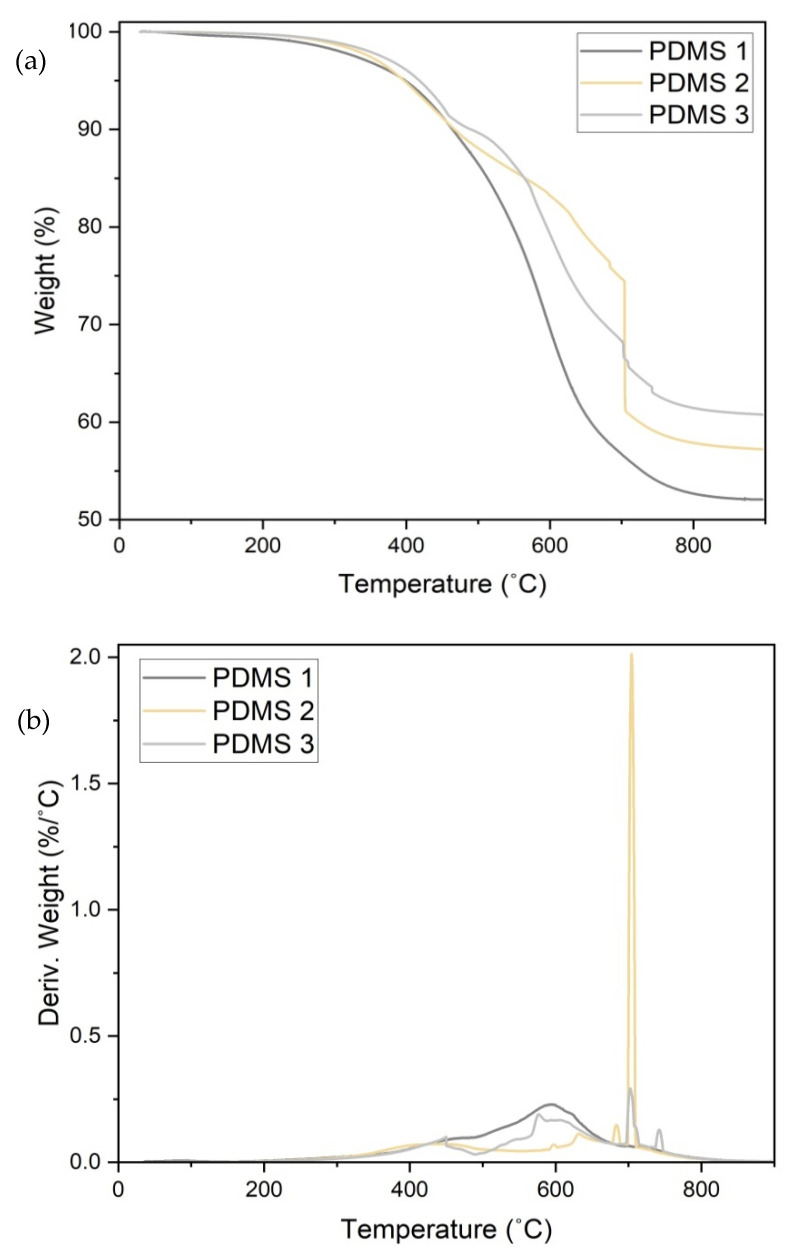
The thermal stability of PDMS 1, PDMS 2, and PDMS 3 (**a**) TGA plot of weight% vs. temperature and (**b**) TGA plot of derivative of weight (%/ C) vs. temperature.

The effect of the iron content on the stability of the materials was evaluated using thermogravimetric analysis (TGA) (Figure 4). PDMS is elastic and stable at temperatures of up to 250 °C. In the present study, the first stage of weight loss (≈1% wt.%.) occurred from 25 °C to 237 °C, which was attributed to the loss of moisture and impurities. The second stage of weight loss occurred from 237 °C to 458 °C, which was attributed to PDMS depolymerization due to the breakage of the Si-O groups in the PDMS chain [24,25]. Above 369 °C, the initial decomposition temperatures of the PDMS 1 and PDMS 2 composites shifted to lower values compared to those for the PDMS 3 composite. PDMS 3 exhibited the highest thermal stability until 554 °C, after which the weight decreased gradually with increasing temperature. It could be that the presence of iron particles improved the constancy of the materials by preventing volatile movement. The final stage of weight loss occurred from 560 °C to 700 °C, which was attributed to the continued decomposition of PDMS. The highest rate of weight loss was observed at 701 °C, as evident from the first derivative peak temperature (Tp) depicted in Figure 4b. The maximum weight loss for PDMS 1, PDMS 2, and PDMS 3 occurred at further higher temperatures of 580 °C, 699 °C, and 701 °C, respectively (Figure 4b). Above 708 °C, PDMS 3 exhibited the highest thermal stability. Finally, the thermal stabilities of the samples stabilized, with final residual amounts of 52%, 57.3%, and 60.8% for PDMS 1, PDMS 2, and PDMS 3, respectively. The residues in the samples were attributed to the presence of inorganic compounds of Fe and Si. PDMS composites with high stability are useful for handling distinct chips for biochemical reactions [26]. In this context, it is important to fabricate uniform and suitably cured PDMS having good thermal stability at elevated temperatures.

### 3.4. SEM Analysis

**Figure 5:**
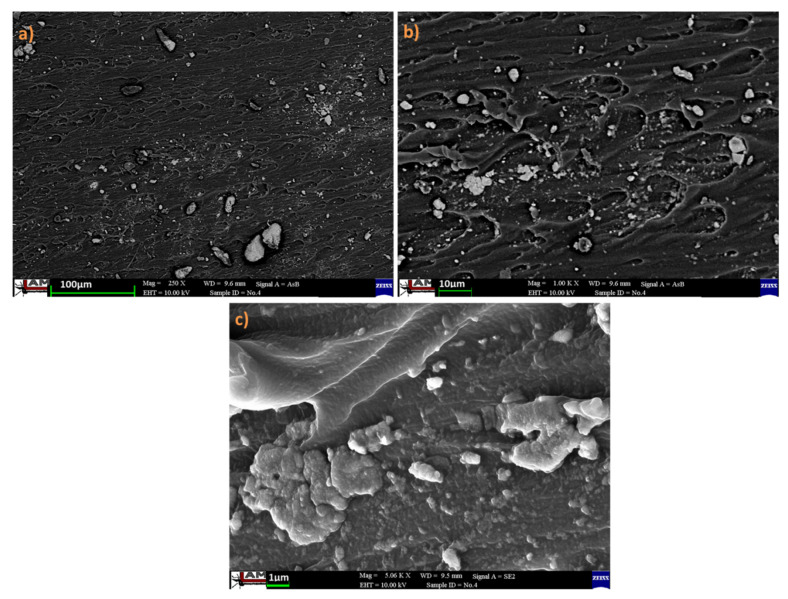
The SEM of PDMS 3 composite at different magnifications (**a**) 250 X (**b**) 1.00 KX and (**c**) 5.06 KX.

Material morphology and the distribution of iron particles in the PDMS 3 composite were investigated using scanning electron microscopy (SEM) (Figure 5). It is noteworthy that, under an applied magnetic field, the iron particles tended to decrease the energy of the magneto-static to reach a stable order. Moreover, iron particles arranged rapidly in a direction parallel to that of the magnetic field. The particles had magnetized in the same tendency, and the magnetic pole interactions between the sphere chains caused the particles to repel each other until an equilibrium interval was reached. Subsequently, the long-chain structure of the particles agglomerated to create columns. These agglomerates were then magnetized under the applied magnetic field and united into linear chains that aligned along the direction of the magnetic field. The chain length was limited by the elastic resistance of the host polymer to the displacement of the agglomerates. The presence of such columns was also detected by Calabro et al. in a previous study [27]. PDMS permits the magnetically responsive particles to respond to the field by deforming the microsphere shape, with the shell extending in the direction of the magnetic field [28,29]. The increase in the temperature using magnetic-induction-based heating leads to appreciable diversity in the structure of materials, and the distribution of the Fe particles in the PDMS matrix affects the properties of the fabricated material. In addition, the agglomeration and orientation of the Fe filler affect the swelling and the mechanical properties of the composites.

### 3.5. Strain–Temperature Properties

**Figure 6:**
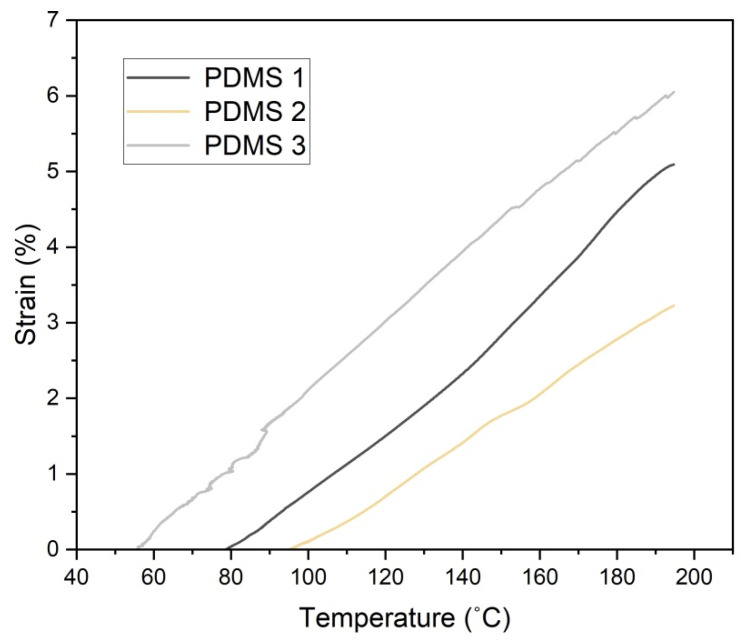
Strain–temperature curve of DMS 1, PDMS 2, and PDMS 3.

The temperature-dependent mechanical properties of the fabricated composites were investigated using dynamic mechanical analysis (DMA). Strain behavior with the increase in temperature for the PDMS composites is illustrated in Figure 6. PDMS 2 exhibited low strain at high temperatures. This was because the considerable interaction between the PDMS matrix and the iron filler limited the heat distortion in the PDMS 2 composite. The strain increased with the increase in the iron content as the distortion in the samples increased with an increase in temperature. PDMS 3 exhibited the highest distortion upon an increase in the temperature due to cluster formation. The filler–filler interactions were governed by electrostatic forces and Van der Waals, which corrupted the mechanical properties of the composite. This was mainly attributed to the difficulty of uniformly dispersing the Fe particles within the polymer matrix and to the incompatibility of Fe with the hydrophobicity of the PDMS matrix. It is reported that at low temperatures, the mechanical property of a material is independent of the heating duration, while at higher temperatures, the mechanical strength is decreased [30].

### 3.6. Swelling Behavior

**Figure 7:**
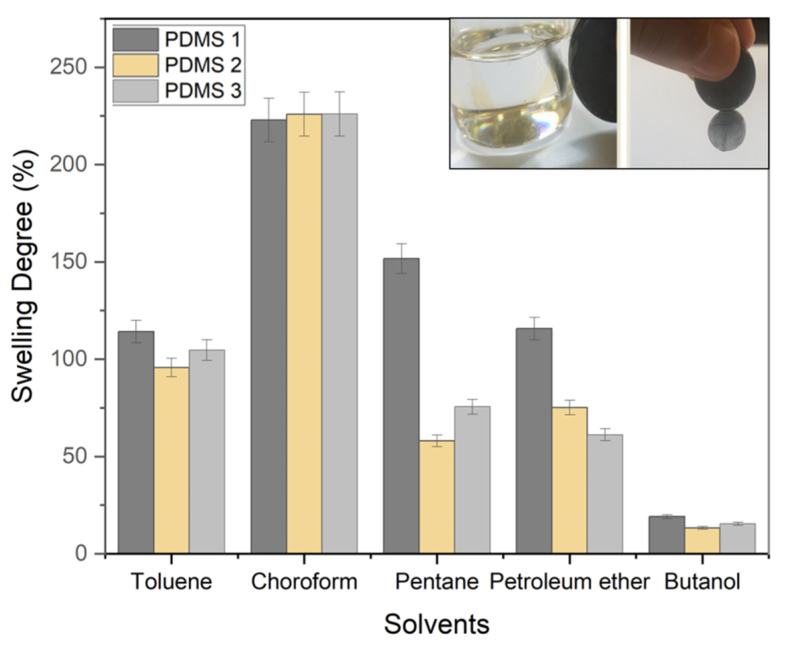
Swelling behavior of the materials in organic solvents and separation by magnetic character.

Volatile organic compounds exert a negative impact on the environment and human health. These compounds evaporate at room temperature and pressure, causing the vapors to be present in both indoor and outdoor environments [3,4,5]. The hazards caused by the increasing amounts of petrochemical solvents, such as toluene, chloroform, pentane, petroleum ether, and n-butanol, should be particularly focused on, as these solvents are highly toxic and difficult to remove from the environment [6,7]. Compound adsorbents, which include shrink-bonded polymers and rubber materials, adsorb fluids on their solid structure and swelling. The PDMS matrix also allows the permeation of organic solvents, leading to an increase in the polymer volume and polymer swelling, which depend on the volume of solvent permeated [31]. The degree of swelling of the material is determined by the solubility of the solvent. The swelling behavior of PDMS in different organic solvents is presented in Figure 7. PDMS swelled to the greatest extent in the presence of chloroform, while a large extent of PDMS swelling was observed in toluene, pentane, and petroleum ether as well. The swelling was the least in n-butanol, which is a polar solvent. In other polar solvents as well, the swelling was less evident. Bhanushali et al. reported that the solvents with δ ≈ 15.5 (MPa)^0.5^ cause the greatest degree of swelling in the PDMS membranes, which have a similar δ value, at a maximum solubility of ~2 g solvent per g of the polymer [32]. A higher degree of swelling could, however, be obtained when the solubility of the solvent was close to that of PDMS. This was attributed to hydrogen-bonding, polarity, and dispersive effects, which depend on the molecular structures and chemical groups of both solvents and polymers. Therefore, the solvents and polymers that have comparable solubility of the parameter are expected to interact strongly and allow the solvent to be soluble in the polymer, thereby allowing considerable polymer swelling to occur. The PDMS swelling observed in the non-polar solvents is due to the empty net structure in the polymer. The degree of swelling might also be affected by the degree of crosslinking, which is affected by the Fe content. The study of the swelling behavior of PDMS in different organic solvents is critical in the case of layout PDMS meant for the extraction process, in which the PDMS composite is used for organic solvent removal based on its magnetic property (Figure 7). The composites that hold solid metal fillers exhibit a range of outstanding properties, such as lower density, low cost, and simple fabrication [33]. These properties, together with the magnetic character, render the composites suitable for application in oil collection [34,35,36]. Magnetic character is also crucial for reusing and separating the composites from the medium simply by using a magnetic field. The materials that could be applied as adsorbents for the removal of organic solvents from the environment using a simple technique are highly desirable.

## 4. Conclusions

The present study investigated the dual functionalities of iron particles as micro-heating agents and fillers in the PDMS matrix. The curing rate was monitored and controlled by altering the Fe content in the system to achieve the required working time. The presence of iron particles enhances the hydrosilation reactivity in a short duration (less than 5 min). Therefore, the magnetic-induction-cured system has easy processability. PDMS is degraded at a low temperature (≈250 °C), while the presence of the iron filler in the PDMS matrix enhances the thermal stability of the material. PDMS 3 exhibited the highest thermal stability among all three composites fabricated. PDMS 2 exhibited enhanced mechanical properties compared to PDMS 1 and PDMS 3. PDMS 2 showed low strain at high temperatures due to the considerable interaction between the PDMS matrix, and the iron filler limited the heat distortion in the PDMS 2 composite. The PDMS composite swelled the most in chloroform, while a large extent of PDMS composite swelling was observed in toluene, pentane, and petroleum ether as well. The swelling was the least in n-butanol. Magnetic PDMS composites are quite efficient and suitable as an adsorbent material for removing organic solvents from the environment.

## Data Availability

Not applicable.
